# *In vivo* Bioluminescent Imaging of Mammary Tumors Using IVIS Spectrum

**DOI:** 10.3791/1210

**Published:** 2009-04-29

**Authors:** Ed Lim, Kshitij D Modi, JaeBeom Kim

**Affiliations:** Biology Research and Development , Caliper Life Sciences

## Abstract

4T1 mouse mammary tumor cells can be implanted sub-cutaneously in nu/nu mice to form palpable tumors in 15 to 20 days. This xenograft tumor model system is valuable for the pre-clinical *in vivo* evaluation of putative antitumor compounds.

The 4T1 cell line has been engineered to constitutively express the firefly luciferase gene (luc2). When mice carrying 4T1-luc2 tumors are injected with Luciferin the tumors emit a visual light signal that can be monitored using a sensitive optical imaging system like the IVIS Spectrum. The photon flux from the tumor is proportional to the number of light emitting cells and the signal can be measured to monitor tumor growth and development. IVIS is calibrated to enable absolute quantitation of the bioluminescent signal and longitudinal studies can be performed over many months and over several orders of signal magnitude without compromising the quantitative result.

Tumor growth can be monitored for several days by bioluminescence before the tumor size becomes palpable or measurable by traditional physical means. This rapid monitoring can provide insight into early events in tumor development or lead to shorter experimental procedures.

Tumor cell death and necrosis due to hypoxia or drug treatment is indicated early by a reduction in the bioluminescent signal. This cell death might not be accompanied by a reduction in tumor size as measured by physical means. The ability to see early events in tumor necrosis has significant impact on the selection and development of therapeutic agents.

Quantitative imaging of tumor growth using IVIS provides precise quantitation and accelerates the experimental process to generate results.

**Figure:**
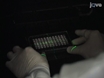


## Protocol

A wide variety of luciferase expressing cancer cell lines can be used for pre-clinical research in mouse models.

These cells are provided as a pathogen-free frozen culture, which will readily grow in standard media with no need for selection markers.

For our experiment, we’ll use the 4T1-luc2 murine mammary tumor cell line, which expresses the luciferase gene that serves as an optical indicator of gene expression or tumorgenesis *in vivo*.  We will use luciferase expression to track growth of the primary tumor non-invasively, but they can also be used to locate and monitor metastatic lesions.

Luciferase activity must be verified before injection, and in order to do this, a 90% confluent flask is harvested by trypsinization and then counted.

50,000 cells are then dispensed in a single well of a microtiter plate and serial dilutions are performed.  Luciferin is added to the wells at 150ug per ml and incubated for 2 minutes.  The microplate can be imaged in IVIS or a luminescent plate reader to determine expression levels.  This cell line expresses up to 6500 photons per second per cell, but any expression level above 500 photons per second per cell can be imaged successfully in vivo.

Now that we have cells of optimal activity, we can proceed to the subcutaneous injection step.

In order to facilitate optimal detection of the tumor, we are using an athymic immunocompromised nude mouse strain.  Prior to injection, animals are anesthetized to effect for deep anesthesia.

Next we will inject up to 250,000 cells in 100ul PBS are injected subcutaneously into the flank.  Load the cells in a 1 ml syringe and attach a 26 gauge needle.  Lift the skin gently with forceps to make a “tent” and inject the cells at the base.   The newly injected cells can be imaged immediately.  In each imaging session a total of 150mg of Luciferin per kg body weight is then administered via two injections into the peritoneal cavity.

In this study, animals are imaged 10 minutes after Luciferin injection to ensure consistent photon flux.  We’ll show you this in the next step.  Luciferin kinetics can vary from day to day.  In this particular model, measuring 10 minutes after Luciferin injection gave a result within a range of 15% variability.

For our experiment we’ll use the IVIS Spectrum in vivo imaging system uses a back-thinned charge coupled device cooled to -90°C to achieve maximum sensitivity.  To support absolute quantitation, the system measures dark charge during down-time and runs a self-calibration during initialization.  To start imaging, initialize the IVIS system (one click) and set the imaging parameters for the experiment.

Select field of view for the number of animals being imaged.  Up to 5 animals can be maintained in the instrument using the integral anesthetic manifold.  The stage is at a constant 37°C to maintain body temperature in the animals.  An EKG port is provided to monitor animal health during stressful procedures.

In Living Image software, exposure time, f-stop and pixel binning can be optimized based on the expression level of the cell line.  These settings can be changed at any time during an experiment without impacting the quantitative result.

IVIS acquires a photographic image of the animal under white light and a quantitative bioluminescent or fluorescent signal, which is overlaid on the image.

The bioluminescent signal is expressed in photons per second and displayed as an intensity map.  The image display is adjusted to provide optimal contrast and resolution in the image without affecting quantitation.

Luminescence from the cells can be measured at the site of injection using a region of interest tool.  Measurement data are displayed in the table together with all experimental parameters relating to the image capture which can be saved or exported for analysis

Multiple images can be acquired and compared in longitudinal studies covering seconds or months depending on the nature of the experiment.  We will measure photon flux from the tumor at time zero and monitor for 4 weeks with imaging at bi-weekly intervals.

Photon flux from the tumor is proportional to the number of live cells expressing luciferase so bioluminescence correlates directly with tumor size.

At 5 days post implantation the tumor is not yet palpable but the cells can be quantified through bioluminescence and the tumor is seen to be actively growing.  At this stage the bioluminescence signal is much stronger.  The exposure time, f-stop and pixel binning can be adjusted so that the image is clear and the camera does not saturate.  IVIS automatically compensates for the changes in light collection so these measurements can be compared to those collected earlier and later in the experiment.

At 7 days post implantation tumors are palpable for the first time and bioluminescence measurement has already generated 7 days of data.

At 28 days post implantation, tumors are becoming necrotic and cells begin to die.  Tumor size estimated by caliper measurement does not change appreciably, but luminescence from the tumor will decrease indicating cell death.

Caliper measurements and bioluminescence measurement can be continued until a humane endpoint is reached.  Tumor necrosis due to hypoxia or treatment regimes will be indicated by reduced bioluminescence even if they do not reduce the tumor mass.

## Disclosures

I would like to assure that all experiments performed to produce the Journal of Visualized Experiments article were performed in compliance with the Animal Protocol 060 approved by the Institutional Animal Care and Use Committee (IACUC) at Caliper Life Sciences, Alameda  CA”  Jae Kim, Ph.D., IACUC Chair, Caliper Life Sciences.

